# Water Physical Exercise Program (WPEP) Using High-Intensity Interval Training in Individuals With Parkinson's Disease: A Clinical Trial Protocol

**DOI:** 10.1155/padi/1946207

**Published:** 2025-04-02

**Authors:** Luís Henrique Paladini, Giovanna Cristina Leveck, Tainá Christinelli, Juliana Siega, André Eduardo Falcoski Doliny, Paulo Cesar Barauce Bento, Vera Lúcia Israel

**Affiliations:** Postgraduate Program in Physical Education, Federal University do Paraná, Curitiba, Paraná, Brazil

## Abstract

**Introduction:** Parkinson's disease (PD) is a chronic, neurodegenerative disease of the central nervous system (CNS). Complications in PD are related to impaired cardiorespiratory capacity, and the presence of motor and nonmotor symptoms, such as reduced cardiorespiratory fitness, decreased respiratory muscle strength and lung volumes and capacities, bradykinesia, muscle rigidity, attenuation of strength and lower limb muscle power, sleep disorders, anxiety, and depressive symptoms. The practice of high-intensity exercise and the examination of the aquatic environment may help to minimize these symptoms and slow disease progression.

**Objective:** To develop a water physical exercise program (WPEP) focusing on high-intensity interval training (HIIT) for individuals with DP.

**Methods:** This is a protocol for a single blinded controlled clinical trial. The sample will consist of individuals with PD between Stages 1 and 4 on the Hoehn and Yahr (HY) Scale, divided into a control group and a WPEP group (which will participate in the WPEP). The outcomes will be divided into three categories: cardiorespiratory, motor, and nonmotor aspects. The WPEP will last 12 weeks, and the intervention will take place two times a week, with a duration of approximately 35 min, with an interval between 48 and 72 h between training sessions for muscle recovery, for a total of 24 sessions. It is expected that this study will establish parameters for prescribing and monitoring a WPEP for individuals with PD Stages 1–4 on the HY scale, enhancing the practice of exercise prescription.

**Trial Registration:** Brazilian Register of Clinical Trials: RBR-3hp5yvh

## 1. Introduction

Parkinson's disease (PD) is the world's fastest growing neurological disease, with biological and socioeconomic consequences [1]. It is also considered a neurodegenerative disease, with chronic and progressive development and multifactorial etiology, involving protein and mitochondrial dysfunctions, degeneration of dopaminergic neurons in the substantia nigra of the mesencephalon, with the influence of genetic, environmental, and age-related factors [2, 3].

Individuals with PD may exhibit motor signs such as bradykinesia, muscle rigidity, resting tremor, and postural instability, in addition to the nonmotor symptoms, ranging from cognition to the functioning of the autonomous nervous system, such as constipation, sexual dysfunction, and sleep disorders, which can directly affect quality of life [4, 5].

Studies have showed that reducing sedentary behavior and increasing the frequency and intensity of physical exercise may help to stabilize and prevent disease progression [6, 7]. In addition, prescription muscle-building and flexibility exercises, as well as proprioceptive and aerobic training are recommended for older adults and individuals with chronic health conditions [8].

Aerobic exercise, especially when prescribed at moderate to high intensity, leads to structural changes in the central and peripheral nervous systems, which lead to brain plasticity and the improvement of body functions and structures in individuals with PD [9, 10]. Thus, the literature has broadened the discussion on high-intensity aerobic exercises for individuals with PD, particularly on high intensity interval training (HIIT), due to its dynamicity, time savings, and prior results equal to or greater than moderate-intensity aerobic training [6, 7, 11].

HIIT is defined by short bouts of high-intensity exercise followed by passive recovery or moderate intensity (active), with running on treadmill or cycling on a ground cycle ergometer being frequently performed activities [7, 12, 13]. Studies have showed the safety of HIIT for individuals with PD, with rare reports of serious adverse events caused to study participants. However, nonsevere adverse effects may still occur, such as falls and pain due to the type, intensity, and speed of the prescribed exercise and the health condition of the person with PD [13–15].

As a result, the execution of aerobic exercises in the aquatic environment emerges as an alternative, as the effect of the heated water's physical properties, such as floatability and viscosity, may improve reaction time and therefore reducing the risk of falls [16–18]. Similarly, the temperature of the water acts on free nerve endings and mechanoreceptors, reducing the conduction of painful stimuli, and increasing blood flow caused by vasodilation. This may provide reduction in muscle tension and joint stiffness, making movement in the water more pleasant and reducing pain perception [16, 18, 19].

Clinical protocol studies are responsible for ensuring standardization of a treatment program, ensuring that the program can be replicated and adapted by other teaching, research, and assistance institutions in a regulated, safe, ethical, and effective manner. To date, we are unaware of any studies that have performed HITT in a heated pool for individuals with PD. Therefore, the objective of this study is to design a program of aquatic physical exercises (WPEP) focusing on HIIT for individuals with PD.

## 2. Materials and Methods

### 2.1. Study Design

This is a protocol for a controlled single blinded clinical trial [20].

The project was approved by the Ethics Committee of the Federal University of Paraná under number 39816320.1.0000.0102. The WPEP study protocol was developed based on the recommendations for clinical trial protocols of the Standard Protocol Items: Recommendations for Interventional Trials (SPIRIT) [21]. This is a high-intensity aquatic physical exercise program to be conducted with individuals with a clinical diagnosis of PD, which will be assessed in two phases, prior to the start of the program and after its completion.

### 2.2. Participants

Individuals with a clinical diagnosis of PD and classified in Stages 1–4 of the Modified Hoehn and Yahr (HY) Scale, residing in the city of Curitiba (PR), of both sexes, and vaccinated against COVID-19 will be invited to participate in the study, with consent to participate in the study voluntarily and sign the free and informed consent term (FICF).

The exclusion criteria will include cognitive, visual, and auditory impairments, as well as the use of wheelchairs (whether due to or not due to PD), cardiovascular surgery, or decompensated cardiac diseases that place the participant at risk, or any absolute contraindication to attend a heated swimming pool, and changes in dosages or parameters of the medication associated with PD during the course of the study [22, 23].

### 2.3. Recruitment

The study will be announced via digital media and at the Parkinson's Association Paraná. A telephone call will be made to potential participants to formalize the invitation to participate and to discuss the eligibility and exclusion criteria with each interested individual. They will also be informed about voluntary participation in the study and the need to sign the FICF.

### 2.4. Assessments

**Figure 1:**
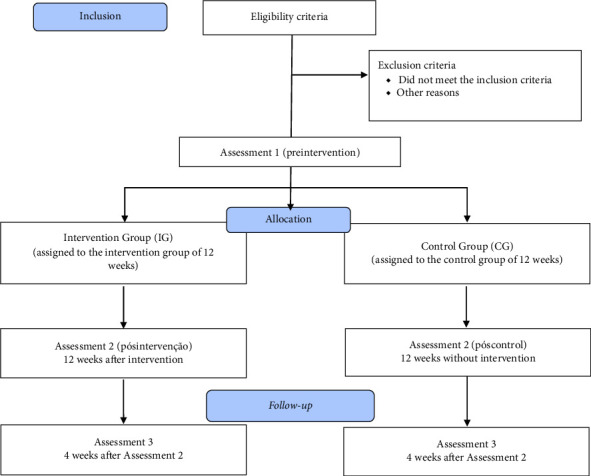
Participant recruitment and allocation.

All assessments will be performed at the “on” stage of dopaminergic medication, approximately 1 h after intake. The assessment instruments will take place in three stages: Assessment 1 (initial), followed by 12 weeks of intervention or control period; Assessment 2, after the intervention period or control of the study; Assessment 3, after a 4-week follow-up (no intervention).  Figure 1 shows the sequence of evaluations and interventions.

### 2.5. Primary Outcomes—Cardiorespiratory Aspects

1. Cardiorespiratory performance—6-minute walking test (6-MWT)
  The 6-MWT is a submaximal test used to indirectly measure cardiorespiratory fitness, functional capacity to exercise, walking distance, and walking observation, and it is a predictor of morbidity and mortality [24]. The incentive phrases and the full test will be conducted according to the guidelines for the 6-MWT of the American Thoracic Society [24].
2. Heart rate variability (HRV)—Heart rate sensor
  The HRV will be collected using a Polar H10 heart rate sensor, which will be placed slightly below the xiphoid process. The participant will remain lying down, quietly, in dorsal decubitus position for 10 min, with spontaneous breathing. The data will be exported in txt format to the *Kubios HRV Standard* 3.4.2 software, where ectopic beats, signs of artifacts, and sinus beats with less than 95% will be excluded from the study [25].
  For the analysis in the time domain, the SDNN values, resulting from the standard deviation of all normal R-R intervals, representing sympathetic and vagal modulation, and the RMSSD, corresponding to vagal modulation, will be obtained by the square root of the mean square of the differences between adjacent normal R-R ranges minus one. The frequency domain is composed of the high frequency (HF), which indicates the activity of the vagus nerve on the heart, the low frequency (LF), which represents both the vagal and sympathetic components on the heart, with predominance of the sympathethic, and the LF/HF ratio that characterizes the sympathovagal balance [26, 27].
3. Pulmonary volumes and capacities—spirometry
  The examination of forced vital capacity (FVC), forced expiratory volume in 1 s (FEV-1), Tiffeneau–Pinelli Index (VEF1/FVC ratio) and maximal voluntary ventilation (MVV) will be performed using a portable spirometer.
4. Maximal inspiratory pressure and maximal expiratory pressure—manuvacuometer
  The maximal inspiratory pressure (MIP) and maximal expiratory pressure (MEP) are correlates of respiratory muscle strength (inspiration: diaphragm and intercostal muscles; expiration: abdominal muscles) and are measured in centimeters of water (cmH_2_0) using manovacuometry [28].

### 2.6. Primary Outcomes—Motor Aspects

1. Activities of daily living (Section 2) and motor symptoms (Section 3)—Unified Parkinson's Disease Rating Scale (UPDRS)
  The UPDRS scale has 4 sections, and in this study only Section 2 and Section 3 will be used, in order to measure the progression of the disease through the participants self-report. Each item is scored from 0 to 4 points, with the higher and lower values corresponding to greater and less disease severity, respectively [29].

### 2.7. Secondary Outcomes—Motor Aspects

1. Lower limbs' strength and power using the five times sit to stand test (5xSTST).
  This test assesses functional mobility, dynamic strength, and muscle endurance of the lower limbs [30]. The execution consists of the participant sitting and rising from a chair, without arm support, as rapidly as possible for five times [30].
2. Lower limb isometric muscle strength—Lafayette
  The quantitative assessment of isometric muscle strength will be performed using a portable dynamometer (Lafayette manual muscle testing system model-01165), which will provide data for peak strength, average strength, and time to peak force. The abductor muscles, hip adductors and flexors, and knee extensors will be assessed [31].
3. Dynamic balance and risk of fall—timed up and go test (TUG)
  The TUG is used to determine mobility and the risk of fall by varying dual cognitive and motor tasks. All three test modes will be performed at the regular speed, in a comfortable and safe manner [32].
4. Static and dynamic balance—mini best test
  The balance evaluation systems test (BESTest), abbreviated as Mini BESTest, will be used to assess changes in static and dynamic postural balance. The test simulates daily activities and retrieves sensory information in some tasks in order to challenge balance [33].

### 2.8. Secondary Outcomes—Nonmotor Aspects

1. Sleep quality—*Parkinson's Disease Sleep Scale* (PDSS)
  The PDSS is a *visual* scale that is used to identify nightly manifestations that may have interfered with sleep in the past week [5].
2. Depressive symptoms—*Geriatric Depression Scale 15* (GDS-15)
  This scale *assesses* nonsomatic symptoms of depression, such as psychological aspects and social implications [34].
3. Quality of life—*Parkinson's Disease Questionnaire 39 (PDQ-39)*
  This instrument *assesses*self-rated quality of life in relation to the previous month, using 39 questions grouped into eight dimensions: mobility, activities of daily living, physical discomfort, communication, emotional well-being, cognition, and stigma regarding PD [23].

### 2.9. Secondary Outcomes—Aquatic Motor Skills

1. Aquatic motor skills—Aquatic Functional Assessment Scale (AFAS)
  The AFAS quantifies the quality of learning for aquatic motor and functional skills, divided into 26 motor behaviors based on motor mastery and independence in water [35, 36].

#### 2.9.1. Medication

All assessments and interventions will be carried out in the “on” period of dopaminergic medication. Any change in the medication prescription will be informed and considered grounds for exclusion from the research.

#### 2.9.2. Allocation

The allocation will be carried out randomly with support from the Sealed Envelope website (https://www.sealedenvelope.com/simple-randomiser/v1/lists). The subjects will be divided into an intervention group and a control group for a 12-week period. Following this period, participants in the control group will have the opportunity to participate in the WPEP.

#### 2.9.3. Intervention Group

##### 2.9.3.1. WPEP

Individuals will participate in the WPEP at the Ana Carolina Moura Xavier Rehabilitation Hospital—Laborer Hospital Complex (CHT-HR), in groups of up to five participants, two times a week, with an approximate duration of 35 min, for 12 weeks, with a period of 48–72 h between training sessions, for muscle recovery, totaling 24 sessions.

The program will be divided into 10 min of warm-up, 15 min of HIIT, and 10 min of cool-down using the Ai-Chi method. Participants will be encouraged to maintain subjective perception of effort above seven on the Borg scale 0–10 [6, 11, 37–40]. In addition, every 4 weeks, the WPEP will assess the progression of the exercises in relation to the load, and every week the intensity, degree of difficulty, and independence in water will be assessed [6, 11, 37–40].  Table 1 shows the exercises that will be used in the WPEP.

The warm-up exercises will be performed at moderate intensity with the main purpose of joint warm up, increase in the heart rate, and social interaction between participants [6, 37, 39, 40]. The main part of the WPEP will consist of HIIT, with multiarticular physical exercises and large muscle groups will be performed [6, 37, 39]. The Borg scale 0–10 will be used to monitor and ensure that the exercises are performed at the intended intensity target [6, 38, 39, 42]. Finally, the cool-down exercise will include the Ai-Chi method with the focus on respiratory and postural control, through progressive bipodal and unipodal exercises [37, 40, 41]. Individual adaptations will be made based on each participant´'s physical and functional characteristics [37, 43].

The program will be conducted during the “on” phase of dopaminergic medication, approximately 1 h after ingestion. In each session, vital sign collection will take place before and after the immersion [11, 39, 40].

The swimming pool will remain between 32 and 33° Celsius, with a depth of 1.10 m and size 10.75 m long by 2.90 m wide.

The exercises will be instructed by a physiotherapist and three students, all with experience in PD and aquatic physiotherapy.

#### 2.9.4. Control Group

Individuals in the control group will be advised to maintain their usual physical activities for 12 weeks while avoiding high-intensity activities and/or swimming in a heated pool during the study period.

#### 2.9.5. Adverse Outcomes

In order to reduce adverse outcomes each participant will be required to have an accompanying person. The staff will comprise professionals and students and will be trained and responsible for assisting research participants during the transition from the ground to the pool and vice versa, as well as monitoring vital signals (arterial blood pressure, heart rate, respiratory rate, and peripheral saturation of O_2_) before and after immersion. The subjective perception of stress will also be monitored, and in case of discomfort or signs of health risk (dizziness, anxiety, muscle and joint pain, and angina), the training will be interrupted and the participant will receive first aid by the research team before being referred to the emergency care unit, if necessary [40].

#### 2.9.6. Sample Size

Based on the prevalence of individuals with PD in Curitiba, a sample calculation was performed using GPower 3.1. As a result, a sample of 30 individuals will be required, with 15 assigned to the intervention group and 15 assigned to the control group.

### 2.10. Statistical Analysis

The data will be analyzed for sphericity and homogeneity with Shapiro–Wilk test. The ANOVA mixed model will be used to compare pre- and postintervention and intergroup comparisons. Afterward, a Bonferroni post hoc analysis will be performed on variables with statistically significant differences to identify which assessments differ from each other. The statistical power will be *p* > 0.05, and the effect size will also be shown. SPSS 20.0 statistical software will be used in all the analyses.

## 3. Discussion

The objective of this study is to develop a WPEP for individuals with PD with the focus on HITT. This study was designed based on the European Directive on Physiotherapy for PD and the Guidelines on Aquatic Therapy for People with PD [37, 43], which encourage the stimulation of each person's potential and functional physical performance while progressively increasing the tolerance to aquatic exercise through moderate to high intensity activities. Furthermore, to specify its effects on the cardiorespiratory, motor, and nonmotor aspects associated with neurodegenerative diseases.

Despite the growing literature on the effects of physical exercise on individuals with PD, the performance of high-intensity terrestrial physical exercises, as well as interval training at high intensity, and the principles of fitness (frequency, type, intensity, and duration) needed to enhance the expected effects in motor, nonmotor, and cardiorespiratory aspects have had limited discussion [6]. We are unaware of any studies that has used continuous or interval physical exercise at high intensity in individuals with PD in an aquatic environment; thus, this is a field that needs further research.

It is currently known that individuals with PD require at least 2 days per week of physical exercise, and 12 weeks is adequate to detect functional changes. It is necessary to investigate long-term programs, since short-period studies, such as 4 weeks, may provide less consistent findings [37, 44].

In terms of duration, sessions between 30 and 60 min are commonly adopted, with short durations, close to 30 min, being sufficient for improvement in health conditions as long as they are performed at high intensity [6, 11, 37, 40].

Regarding dose, much is debated regarding the need for individuals with PD, particularly in the early stages, to perform activities with vigorous intensities, and the intensity achieved will depend on the clinical stage, age, and previous experiences with physical exercises [6, 11, 37, 40, 43]. In addition, there is a lack of assessments evaluating the intensity of aquatic physical exercises to prevent the execution of underestimated or overestimated movements [11, 42, 43].

From this, intensity monitoring based on HR may be described, provided that the physical properties of heated water are taken into account, which provide reduction in HR when compared to a nonaquatic environment (e.g., soil) [18, 45]. This occurs due to the apparent weight reduction that is caused according to the Archimedes principle, the buoyancy, and that, associated with hydrostatic pressure, is responsible for increasing venous return and generating hypervolemia in the chest cavity and heart. Therefore, it is necessary to consider these changes in the prescription of exercise, as well as the function of the autonomic nervous system and cardiovascular system of the person with PD and the use of drugs, such as beta-blockers. Kruel et al. [46] suggest a liquid medium adapted formula in which the delta (variation) of heart rate = heart rate in orthostatic position on the ground − heart rate in orthostatic position in immersion at the height of the xiphoid process. The 0–10 or 6–20 Borg scales which assess the subjective perception of exertion are also viable options, as they are easy to apply and cost effective, as long as individuals are trained to understand the scale [38].

In addition to beta-blockers, we considered the use of dopaminergic medication, such as Levodopa, considered the main pharmacological option for PD treatment and responsible for reestablishing dopamine levels. All assessments and intervention were carried out in the “on” phase of the medication, approximately 1 h after ingestion to make the best use of the medication action and the therapeutic window. However, long-term use of dopaminergic medication can cause adverse effects such as dyskinesias, drowsiness, and postural hypotension. Thus, the association between dopaminergic medication and moderate- to high-intensity physical exercise can be considered the gold standard for stabilizing the disease and alleviating unwanted symptoms [6, 42].

Studies that used the interval training methodology in individuals with PD performed on the ground employed activities that used treadmill and cycle ergometer [7, 11, 12, 15] with diversified parameterization. Malczynska-Sims et al. [15] performed 10 stages of 4 min, with 2 min for cycling in the fast stage and 2 min for the slow stage, and the training range was individualized and progressive according to the weeks and the maximum heart rate (60%–80%). Fernandes et al. [7] employed walking/jogging for 25 min with 1 min in the subjective perception of exertion 15–17 and 2 min between 11 and 13. Harvey et al. [11] conducted a feasibility study for HIIT using circuit dynamics and functional exercises, with four exercises performed at 85% of the maximal heart rate for 45 and 15 s of interval/rest without severe adverse effects. All these previous studies showed that HIIT on the ground is a safe modality for individuals with PD, in terms of adherence, participation, and increase in the physical and functional capacity. These findings may be used as a basis for interval training in an aquatic environment, since the literature on this subject is limited.

Finally, this WPEP was developed to assess this type of clinical intervention in PD population in an aquatic setting. The present clinical trial may have the following limitations: (1) the difficulty in recruiting and adhering to the study for 12 weeks plus the pre- and postintervention evaluation period; (2) the inability of participants to travel to the center where the study will be conducted, particularly Stage 4 participants; (3) difficulty in reaching target intensity due to changes in the autonomic nervous system and heterogeneity of the PD population. Furthermore, the WPEP does not include the use of an individual aquatic heart rate monitor (e.g., an aquatic heart rate monitor) during the intervention period to detect changes in the heart rate during immersion. All these limitations must be considered in future research.

However, details in the description of training and its prescription based on individuality and periodic progression, as well as easiness to apply and cost-effective assessments, may facilitate the WPEP clinical reproducibility.

Given the detailed description of this research protocol, if the WPEP provides positive results in terms of the evolution of PD symptoms through the assessed aspects, future studies using this methodology may be conducted to assist and guide the clinical practice of other health professionals who work with this population.

## 4. Conclusion

Based on the biopsychosocial model of health, this study aims to establish prescription parameters and progression of high-intensity aquatic physical exercise for people with PD.

## Figures and Tables

**Table 1:** Training program with exercise description and progress.

Aquatic exercises and progression	Prescription and progression
• Knee flexion in orthostatic position with parallel bar support • Stationary race with or without parallel bar support • Pull apart (shoulder horizontal abduction and adduction) • Leg flexion and extension with grab rail support participant in supine position • Frontal jumping jacks	Weeks 1-2: [6, 11, 39] Total of five exercises per round, being executed in two rounds: - 15 s of adaptation (slow to moderate execution) - 30 s of intense (rapid execution) - 45 s of resting (active resting and swimming pool walking)
• Knee flexion associated with UL • Stationary race without support/running in the swimming pool • Pull apart (shoulder horizontal abduction and adduction) with 1 to 2 kg dumbbells • Leg flexion and extension—participants in prone position • Frontal jumping jacks with 1 to 2 kg dumbbells	Weeks 3–7: [6, 11, 39] Total of five exercises per round, being executed in two rounds: - 45 s intense (rapid execution) - 45 s of resting (active resting and swimming pool walking)
• Ankle reach (UL touches the opposite LL) • Swimming pool running with aquafins • Pull apart (shoulder horizontal abduction and adduction) with 1-2 kg dumbbells associated with LL abduction • Adapted backstroke (as seen in the participant's environment) with aqua tube support and/or the therapist's support • Frontal jumping jacks with 1 to 2 kg dumbbells	Weeks 8–12: [6, 11, 39] Total of five exercises per round, being executed in two rounds: - 45 s intense (rapid execution) - 45 s of resting (active resting and swimming pool walking)

**Warm-up**	**Cool-down**

Swimming pool walk and group ludic exercises to increase heart rate, warm-up of the joints, and social interaction among participants [6, 11, 39]	Ai-Chi method relaxation and breathing control [39, 41] Exercises 1 (contemplating), 2 (fluctuating), and 3 (elevating) in Weeks 1-2 Exercises 4 (closing), 5 (crossing), and 6 (calming) in Weeks 3–7 Exercises 7 (grouping), 8 (freeing), 9 (accepting), 10 (accepting with grace), and 11 (circling) in Weeks 8–12

Abbreviations: kg = kilograms; LL = lower limbs; UL = upper limbs.

## Data Availability

Underlying data may be requested from the research authors.
